# Supplementary material for: Salvadoran Celastraceae Species as a Source of Antikinetoplastid Quinonemethide Triterpenoids
Source: Plants (Basel). 2024 Jan 25;13(3):360. doi: 10.3390/plants13030360 (PMC10857229; doi:10.3390/plants13030360)
Supplement: Supplementary file 1 [file plants-13-00360-s001.zip › plants-2784014-supplementary.pdf]

Electronic Supporting Information

Salvadoran Celastraceae species as a source of antikinetoplastid quinomethide triterpenoids

Marvin J. Núñez[1], Morena L. Martínez[1], Ulises G. Castillo[1], Karla Carolina Flores[1], Jenny Menjívar[2], Atteneri López-Arencibia[3,4,5], Carlos J. Bethencourt-Estrella[3], Ignacio A. Jiménez[6], José E. Piñero[3,4,5], Jacob Lorenzo-Morales[3,4,5] and Isabel L. Bazzocchi[6,*]

[1] Laboratorio de Investigación en Productos Naturales (LIPN), Facultad de Química y Farmacia, Universidad de El Salvador, Final Ave. Mártires Estudiantes del 30 de Julio, 01101, San Salvador, El Salvador
[2] Museo de Historia Natural de El Salvador, Ministerio de Cultura, Final Calle Los Viveros, Col. Nicaragua, 01101, San Salvador, El Salvador
[3] Instituto Universitario de Enfermedades Tropicales y Salud Pública de Canarias, Universidad de La Laguna, 38200 La Laguna, Tenerife, Spain
[4] Departamento de Obstetricia y Ginecología, Pediatría, Medicina Preventiva y Salud Pública, Toxicología, Medicina Legal y Forense y Parasitología, Universidad de La Laguna, 38200 La Laguna, Tenerife, Spain
[5] Centro de Investigación Biomédica en Red de Enfermedades Infecciosas (CIBERINFEC), Instituto de Salud Carlos III, 28220 Madrid, Spain
[6] Instituto Universitario de Bio-Orgánica Antonio González, Departamento de Química Orgánica, Universidad de La Laguna, Avenida Astrofísico Francisco Sánchez 2, 38206 La Laguna, Tenerife, Spain

Table of Contents

Table S1: Organic extracts and fractions of Celastraceae species assayed as anti-kinetoplastid against *Trypanosoma cruzi*, *Leishmania amazonensis*, and *Leishmania donovani.*

Figure S1. Selected Thin Layer Chromatography (TLC) of phytochemical analysis.

Table SI. Organic extracts and fractions of Celastraceae species assayed against T*rypanosoma cruzi*, *Leishmania amazonensis*, and *Leishmania donovani*.

<table>
<tr><th>Specie</th><th>Plant part</th><th>Fraction or Extract</th><th>T. cruzi IC_{50} (µg/mL)</th><th>L. amazonensis IC_{50} (µg/mL)</th><th>L. donovani IC_{50} (µg/mL)</th></tr>
<tr><td rowspan="10">Maytenus segoviarum</td><td rowspan="2">Leaves</td><td>F/DCM</td><td rowspan="4">inactive</td><td rowspan="2">inactive</td><td rowspan="4">inactive</td></tr>
<tr><td>F/n-BuOH</td></tr>
<tr><td rowspan="2">Branches</td><td>F/DCM</td><td>7.83 ± 0.33</td></tr>
<tr><td>F/n-BuOH</td><td>inactive</td></tr>
<tr><td rowspan="3">Root bark</td><td>E/hex:Et_2O (1:1)</td><td>1.36 ± 0.17</td><td>0.85 ± 0.10</td><td>>100</td></tr>
<tr><td>E/MeOH</td><td>>100</td><td>36.50 ± 4.49</td><td rowspan="9">inactive</td></tr>
<tr><td>E/Acetone</td><td>>50</td><td>>50</td></tr>
<tr><td rowspan="3">Fruits</td><td>E/hex:Et_2O (1:1)</td><td rowspan="7">inactive</td><td rowspan="3">inactive</td></tr>
<tr><td>E/MeOH</td></tr>
<tr><td>E/Acetone</td></tr>
<tr><td rowspan="10">Quetzalia ilicina</td><td rowspan="2">Leaves</td><td>F/DCM</td><td>15.20 ± 0.80</td></tr>
<tr><td>F/n-BuOH</td><td>inactive</td></tr>
<tr><td rowspan="2">Branches</td><td>F/DCM</td><td>>50</td></tr>
<tr><td>F/n-BuOH</td><td>inactive</td></tr>
<tr><td rowspan="3">Root bark</td><td>E/hex:Et_2O (1:1)</td><td>3.45 ± 0.35</td><td>3.06 ± 0.77</td><td>>50</td></tr>
<tr><td>E/MeOH</td><td>3.06 ± 0.68</td><td>1.40 ± 0.21</td><td>>100</td></tr>
<tr><td>E/Acetone</td><td>1.58 ± 0.03</td><td>1.34 ± 0.22</td><td>5.42 ± 0.85</td></tr>
<tr><td rowspan="3">Fruits</td><td>E/hex:Et_2O (1:1)</td><td rowspan="7">inactive</td><td rowspan="3">inactive</td><td>inactive</td></tr>
<tr><td>E/MeOH</td><td>51.76 ± 0.95</td></tr>
<tr><td>E/Acetone</td><td rowspan="5">inactive</td></tr>
<tr><td rowspan="7">Zinowiewia integerrima</td><td rowspan="2">Leaves</td><td>F/DCM</td><td>>50</td></tr>
<tr><td>F/n-BuOH</td><td>inactive</td></tr>
<tr><td rowspan="2">Branches</td><td>F/DCM</td><td>23.78 ± 0.44</td></tr>
<tr><td>F/n-BuOH</td><td>inactive</td></tr>
<tr><td rowspan="3">Root bark</td><td>E/hex:Et_2O (1:1)</td><td>0.71 ± 0.04</td><td>0.59 ± 0.05</td><td>>100</td></tr>
<tr><td>E/MeOH</td><td>2.87 ± 0.39</td><td>1.13 ± 0.07</td><td>>100</td></tr>
<tr><td>E/Acetone</td><td>0.75 ± 0.07</td><td>0.38 ± 0.08</td><td>>100</td></tr>
<tr><td rowspan="10">Wimmeria cyclocarpa</td><td rowspan="2">Leaves</td><td>F/DCM</td><td rowspan="4">inactive</td><td rowspan="4">inactive</td><td rowspan="4">inactive</td></tr>
<tr><td>F/n-BuOH</td></tr>
<tr><td rowspan="2">Branches</td><td>F/DCM</td></tr>
<tr><td>F/n-BuOH</td></tr>
<tr><td rowspan="3">Root bark</td><td>E/hex:Et_2O (1:1)</td><td>>50</td><td>6.96 ± 0.88</td><td>>50</td></tr>
<tr><td>E/MeOH</td><td>inactive</td><td>24.99 ± 0.74</td><td>inactive</td></tr>
<tr><td>E/Acetone</td><td>>50</td><td>9.32 ± 1.96</td><td>>50</td></tr>
<tr><td rowspan="3">Fruits</td><td>E/hex:Et_2O (1:1)</td><td rowspan="11">inactive</td><td rowspan="7">inactive</td><td rowspan="11">inactive</td></tr>
<tr><td>E/MeOH</td></tr>
<tr><td>E/Acetone</td></tr>
<tr><td rowspan="8">Euonymus enantiophyllus</td><td rowspan="2">Leaves</td><td>F/DCM</td></tr>
<tr><td>F/n-BuOH</td></tr>
<tr><td rowspan="2">Branches</td><td>F/DCM</td></tr>
<tr><td>F/n-BuOH</td></tr>
<tr><td rowspan="3">Root bark</td><td>E/hex:Et_2O (1:1)</td><td>2.05 ± 0.10</td></tr>
<tr><td>E/MeOH</td><td>precipitate</td></tr>
<tr><td>E/Acetone</td><td>4.58 ± 0.05</td></tr>
<tr><td>Fruits</td><td>E/Acetone</td><td>inactive</td></tr>
</table>

E= extract; F= fraction; DCM = Dichloromethane; n-BuOH = n-butanol; hex = hexanes; inactive > 100 µg/mL.

Figure SI. Selected Thin Layer Chromatography (TLC) of phytochemical analysis.

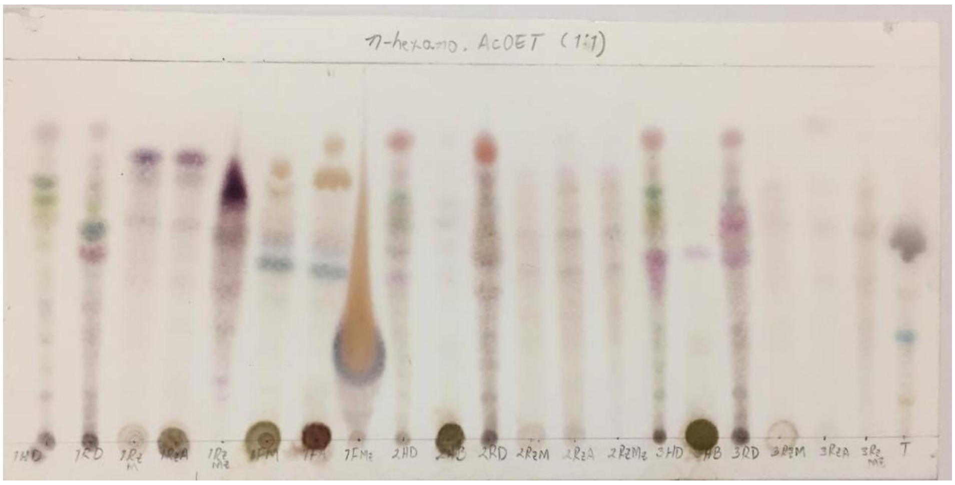

<u>Sterols TLC </u>[hexanes-ethyl acetate (1:1) developed with Liebermann-Burchard's reagent]

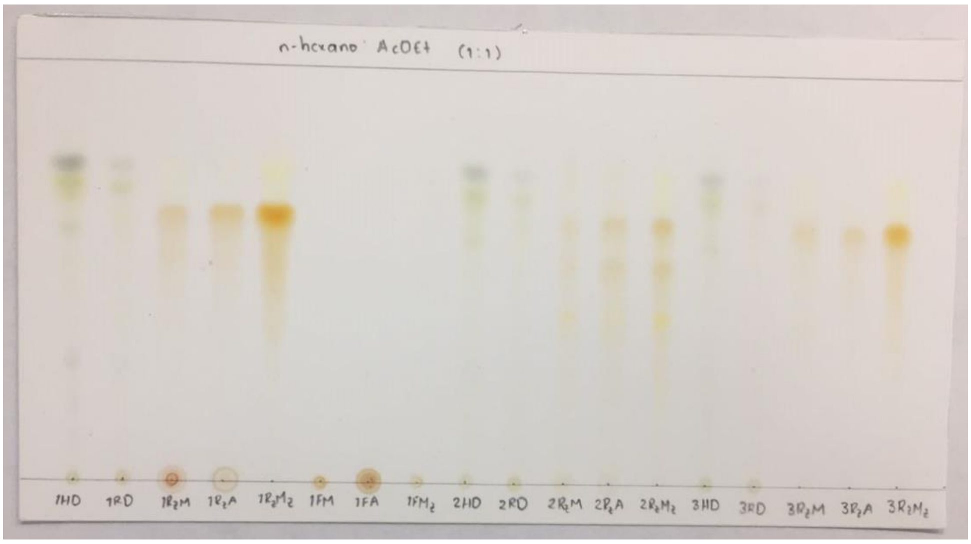

<u>QMTs TLC</u> [(hexanes-ethyl acetate (1:1); developed with visible light]

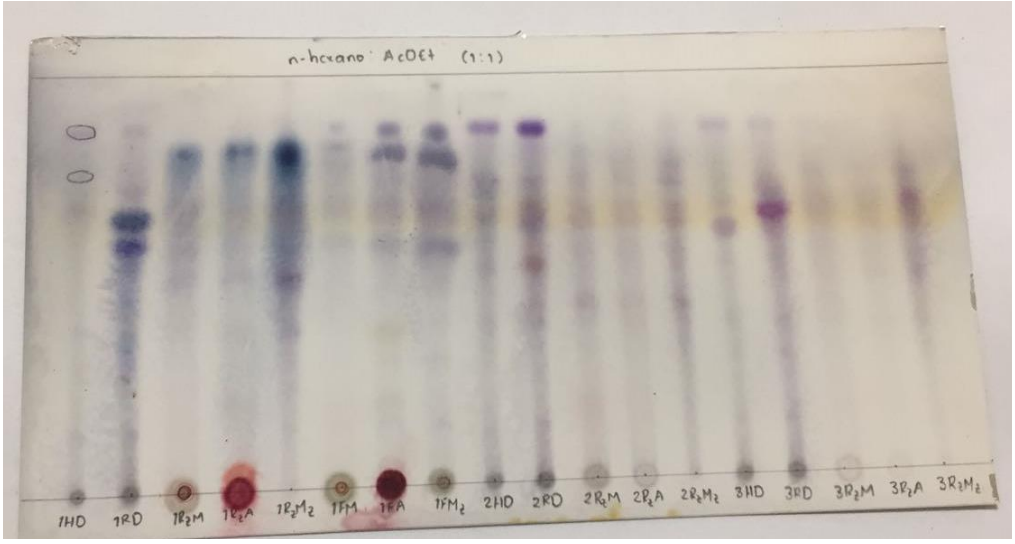

<u>QMTs TLC</u> [(hexanes-ethyl acetate (1:1); developed with Oleum's reagent and heat 100°C]

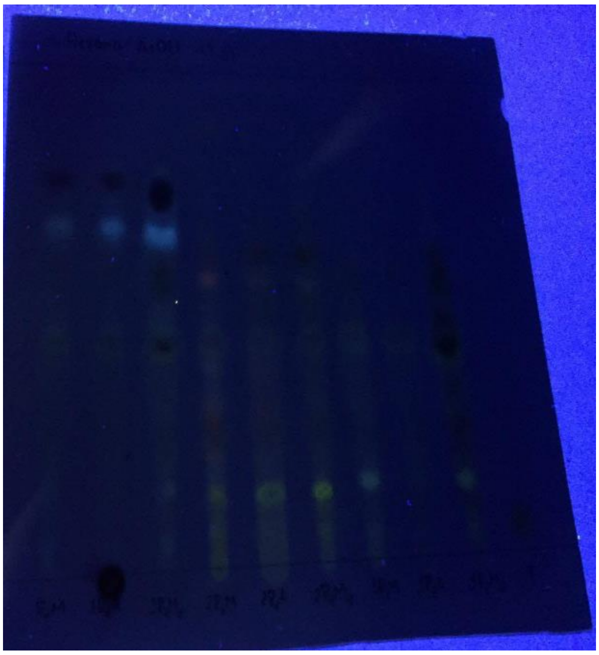

Flavonoids TLC [(hexanes-ethyl acetate (1:1); developed with 1% aluminum trichloride's reagent, and UV-365 nm]

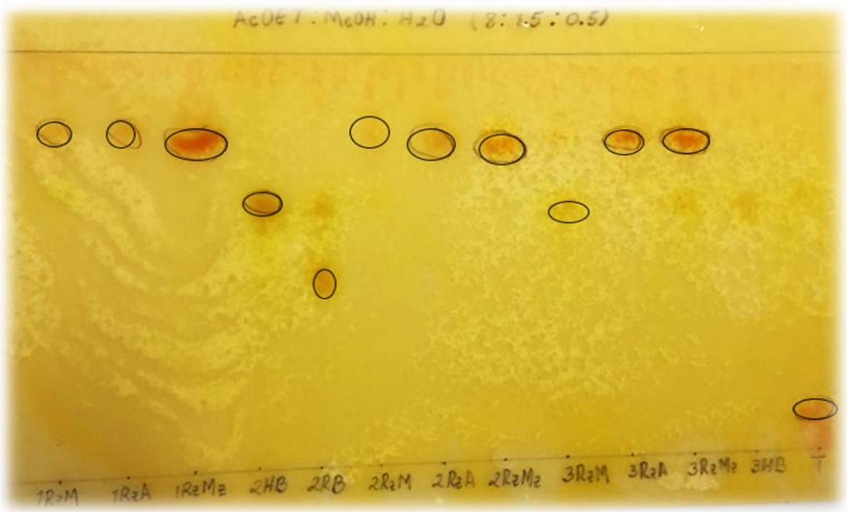

Alkaloids TLC [(ethyl acetate-MeOH-H_2O (8:1.5:0.5); developed with Dragendorff's reagent]

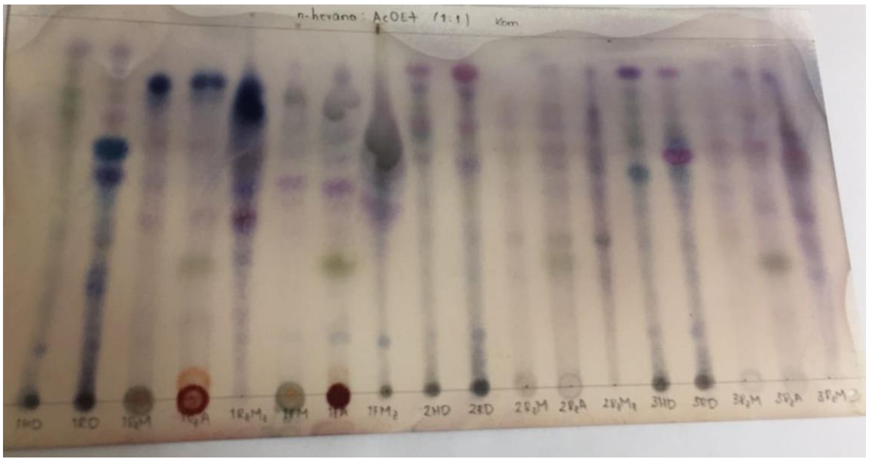

Triterpenes TLC [(hexanes-ethyl acetate (1:1); developed with Vainillin-sulfiric acid's reagent and heat 100°C]
